# Listeria monocytogenes Traffics from Maternal Organs to the Placenta and Back

**DOI:** 10.1371/journal.ppat.0020066

**Published:** 2006-06-30

**Authors:** Anna I Bakardjiev, Julie A Theriot, Daniel A Portnoy

**Affiliations:** 1 Department of Molecular and Cell Biology, University of California Berkeley, Berkeley, California, United States of America; 2 Department of Biochemistry, Stanford University Medical Center, Stanford, California, United States of America; 3 Department of Microbiology and Immunology, Stanford University Medical Center, Stanford, California, United States of America; 4 School of Public Health, University of California Berkeley, Berkeley, California, United States of America; Tufts University School of Medicine, United States of America

## Abstract

Infection with Listeria monocytogenes is a significant health problem during pregnancy. This study evaluates the role of trafficking between maternal organs and placenta in a pregnant guinea pig model of listeriosis. After intravenous inoculation of guinea pigs, the initial ratio of bacteria in maternal organs to placenta was 10^3^–10^4^:1. Rapid increase of bacteria in the placenta changed the ratio to 1:1 after 24 h. Utilizing two wild-type strains, differentially marked by their susceptibility to erythromycin, we found that only a single bacterium was necessary to cause placental infection, and that L. monocytogenes trafficked from maternal organs to the placenta in small numbers. Surprisingly, bacteria trafficked in large numbers from the placenta to maternal organs. Bacterial growth, clearance, and transport between organs were simulated with a mathematical model showing that the rate of bacterial clearance relative to the rate of bacterial replication in the placenta was sufficient to explain the difference in the course of listeriosis in pregnant versus nonpregnant animals. These results provide the basis for a new model where the placenta is relatively protected from infection. Once colonized, the placenta becomes a nidus of infection resulting in massive reseeding of maternal organs, where L. monocytogenes cannot be cleared until trafficking is interrupted by expulsion of the infected placental tissues.

## Synopsis

Listeria monocytogenes is a bacterial pathogen that can cause invasive disease in predisposed individuals, including pregnant women and immunocompromised individuals. During pregnancy, listeriosis leads to spontaneous abortion, preterm labor, or neonatal disease. Tropism of L. monocytogenes to the placenta and maternal immunosuppression, have been hypothesized to be the cause of the susceptibility to listeriosis during pregnancy. This study presents a series of experiments in a pregnant guinea pig model of listeriosis and mathematical simulation of the infection, which led the authors to propose a new model. A single bacterium is sufficient to cause placental infection. Due to decreased clearance in the placenta there is a strong increase of bacteria in the placental compartment, which becomes a nidus of infection leading to continuous seeding of maternal organs. Thus, the increase of bacteria in maternal organs is not due to immunosuppression but to efflux of L. monocytogenes from the placenta. This process will be interrupted by expulsion of the infected feto-placental tissues. Therefore, spontaneous abortion and prematurity can be regarded as survival mechanisms for the mother. Furthermore, this study hypothesizes that expulsion of the infected placenta may be important for the natural history of listeriosis.

## Introduction

Listeriosis during pregnancy can lead to intrauterine infection resulting in severe complications such as preterm labor, spontaneous abortion, stillbirth, or infection of the neonate resulting in high morbidity and mortality [1,2]. Despite its clinical importance, little is known about the molecular and cellular mechanisms leading to placento-fetal infection, or the role of pregnancy in the development of listeriosis. One possible explanation for the increased susceptibility to listeriosis during pregnancy has been sought in the unique immunological condition of mammalian reproduction, where the maternal immune system tolerates paternal alloantigens expressed in fetal tissues. In 1953, Peter Medawar formulated possible mechanisms to explain the immunological paradox of pregnancy, including immunological indolence or inertness of the mother [3]. Since then, pregnancy has been regarded as a state of immunosuppression; in particular, of the cell-mediated arm of the immune system [4,5]. A decrease in cell-mediated immunity could explain an increased susceptibility to infection with the facultative intracellular bacterial pathogen, *Listeria monocytogenes;* and, in fact, has been postulated as the reason for increased incidence of listeriosis during pregnancy [6]. However, the extent and exact nature of modification of the maternal immune system during pregnancy and its contribution to susceptibility to infection still remains largely unknown [7].

Infection of humans and other mammals with L. monocytogenes has been traced to contaminated foods. Once ingested, L. monocytogenes is able to cross the intestinal barrier [8]. Invasive disease is thought to occur secondary to hematogenous dissemination and typically leads to infection of the placento-fetal unit during pregnancy or to meningitis in immunocompromised individuals [9]. In addition to its clinical importance, L. monocytogenes is very amenable to experimental analysis, and its genetics, cellular biology, and pathogenesis have been studied extensively over the past two decades [10,11].

We have previously developed a pregnant guinea pig model of listeriosis [12]. We chose the pregnant guinea pig as our model because both guinea pigs and humans have a hemomonochorial placenta and are susceptible to placento-fetal infection with a variety of pathogens including cytomegalovirus [13,14]. Mice are not susceptible to placento-fetal infection with cytomegalovirus [15,16]. Listeriosis in the pregnant mouse model can only be induced with high infectious inocula leading to significant morbidity and mortality of the mother [17]. We therefore used the pregnant guinea pig and were able to show that extracellular L. monocytogenes does not have a strong tropism to the guinea pig placenta, but once infected, L. monocytogenes cannot be eliminated effectively from the placenta and subsequently spreads from the placenta to the fetus [18]. Importantly, there is a rapid increase in the number of bacteria in the placenta, which is unparalleled in any maternal organs. The rise of placental bacteria over the course of infection could be due to increased replication, decreased clearance, or influx of L. monocytogenes from maternal organs.

In this study we present evidence that L. monocytogenes traffics from maternal organs to the placenta. Interestingly, the influx of bacteria to the placenta occurred in very small numbers, and only a single bacterium was sufficient to cause placental infection. Surprisingly, L. monocytogenes trafficked from the placenta back to maternal organs in large numbers, and the presence of an infected placenta was sufficient to explain the difference in the course of listeriosis in pregnant versus nonpregnant animals. Thus, the most striking finding of this study was that lack of bacterial clearance in the placenta, and not systemic suppression of the maternal immune response, was responsible for the pathogenesis of listeriosis during pregnancy.

## Results/Discussion

### Differences in the Course of Infection between Pregnant and Non-Pregnant Animals

**Figure 1:**
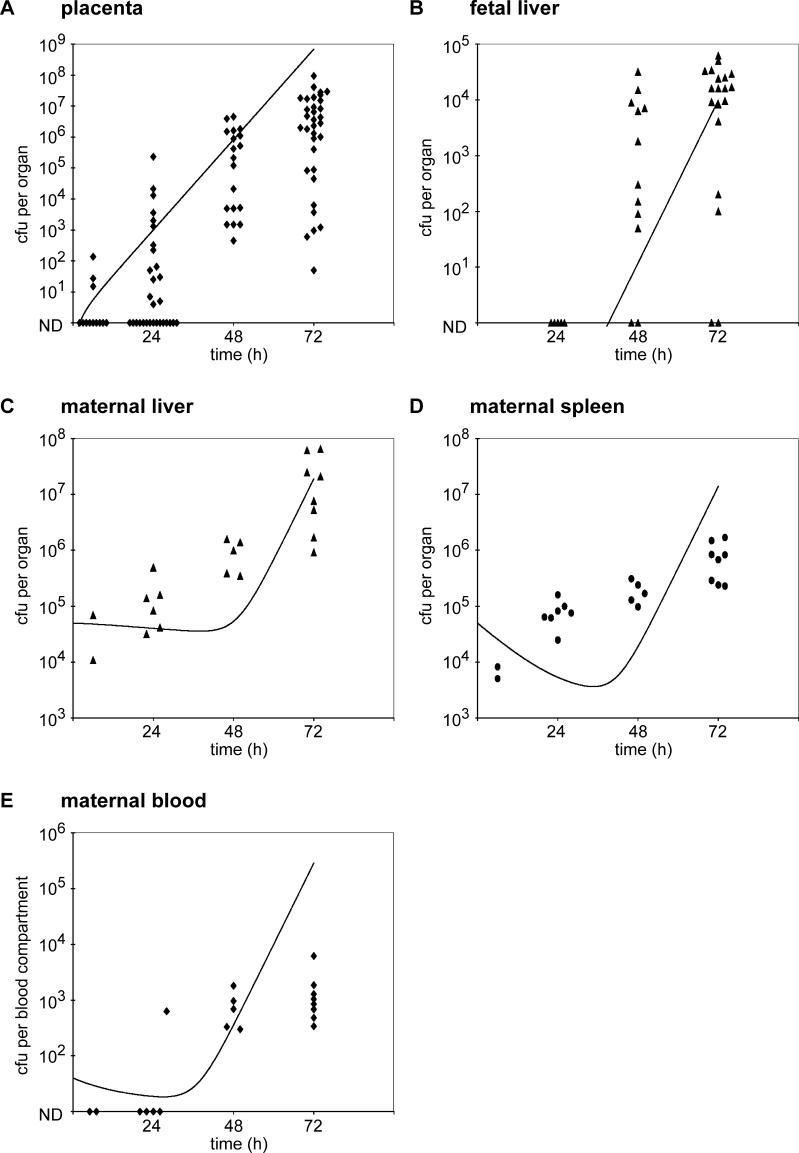
Listeriosis in Pregnant Guinea Pigs and Mathematical Simulation of the Infection CFUs per (A) individual placentas (on average 3–4 per animal), (B) fetal liver, maternal (C) liver, (D) spleen, and (E) blood were enumerated 6, 24, 48, and 72 h after i.v. inoculation of animals with 7.5 × 10^5^–10^6^ wild-type L. monocytogenes. 3/11 animals had no placental infection 72 h p.i. and were excluded from this figure. CFUs for maternal organs in pregnant animals without placental infection are shown in Figure S1. The black line represents the mathematical simulation of CFUs per compartment. ND = not detectable.

**Figure 2:**
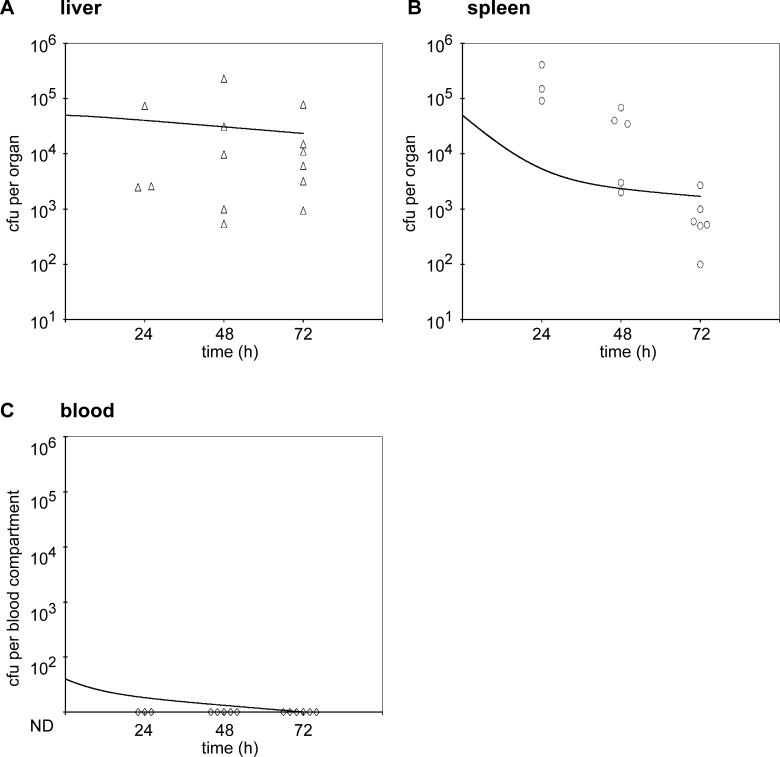
Listeriosis in Nonpregnant Guinea Pigs and Mathematical Simulation of the Infection CFUs per (A) liver, (B) spleen, and (C) blood were enumerated 24, 48, and 72 h after i.v. inoculation of animals with 7.5 × 10^5^–10^6^ wild-type L. monocytogenes. The black line represents the mathematical simulation of CFUs per compartment. ND = not detectable.

In order to understand the effect of pregnancy on the pathogenesis of listeriosis, we compared the bacterial load in maternal organs between pregnant and nonpregnant animals. In addition, we wanted to examine the role of trafficking of L. monocytogenes from maternal organs to the placenta. Previous results showed that the placenta is seeded by 10^3^- to 10^4^-fold lower numbers than maternal spleen and liver immediately after intravenous (i.v.) inoculation with L. monocytogenes [18]. In order to be able to evaluate trafficking from maternal organs to the placenta, we titrated the inoculum of L. monocytogenes to find a dose that would lead to robust initial seeding of maternal liver and spleen, without seeding of the placenta. Injection of pregnant guinea pigs with 7.5 × 10^5^–10^6^ bacteria led to infection of only 3/12 placentas at 6 h post-inoculation (p.i.), and the infected placentas contained 100 or less colony forming units (CFUs) (Figure 1). At the same time, the maternal liver and spleen were found to contain around 10^4^ CFUs (Figure 1). The dose of 7.5 × 10^5^–10^6^ bacteria was used to compare infection in pregnant versus nonpregnant animals. In nonpregnant animals, bacteria were cleared from the spleen, showed no change in the liver, and could not be detected in the bloodstream over the 3-d course of infection (Figure 2). Pregnant animals, on the other hand, were unable to clear bacteria from the liver and spleen and were found to have about 10^3^-fold higher CFUs in both organs at 72 h p.i. than nonpregnant animals (compare Figures 1 and 2). In addition, all pregnant animals were bacteremic at 48 and 72 h p.i. (Figure 1). Interestingly, 3/11 pregnant animals did not have infected placentas 72 h p.i. These animals were not bacteremic and had similar CFUs in the liver and spleen as nonpregnant animals (compare Figures 2 and S1). In animals with placental infection, the bacterial load in the placenta increased by a median of 10^7^-fold and the fetuses became infected over the 3-d course of infection (Figure 1).

### Placental Infection Can Arise from a Single Bacterium

**Figure 3:**
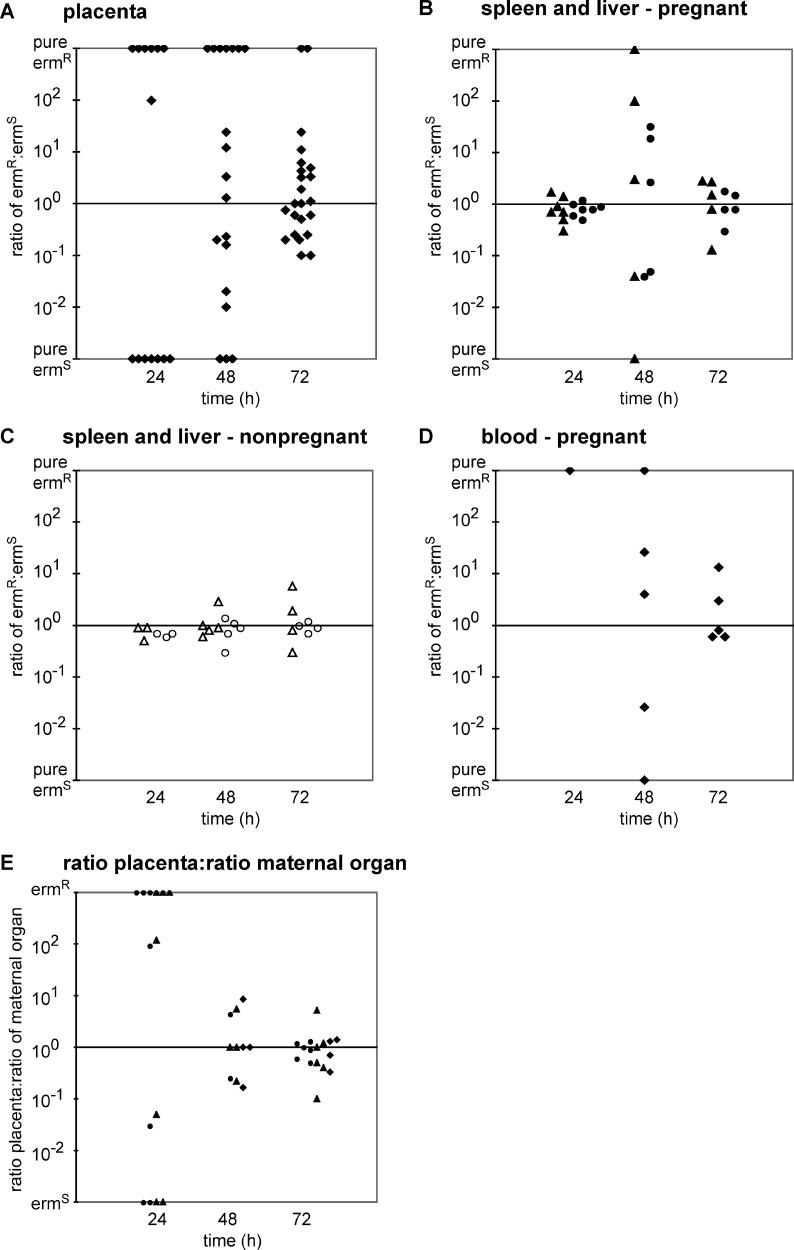
Trafficking of L. monocytogenes in Guinea Pigs Guinea pigs were inoculated i.v. with a 1:1 mixture of 10403S and DP-L3903 at a total dose of 7.5 × 10^5^–10^6^. Ratios between erm^R^ and erm^S^ CFUs from individual placentas (A), maternal liver (triangle), and spleen (circle) of pregnant (B) and nonpregnant (C), and maternal blood (D) of pregnant animals were calculated at 24, 48, and 72 h p.i.. In (A–D) “pure erm^R^” or “pure erm^S^” represents pure cultures of the erm^R^ or erm^S^ strain, respectively. (E) Ratio of placental ratio (pooled placentas from each animal) and ratio of maternal spleen (circle), liver (triangle), and blood (diamond). In (E) “erm^R^” and “erm^S^” represents pure cultures of the respective strains in the placenta and a ratio around 1 in maternal organs. Data points with pure cultures of the same strain in placenta and maternal organs were plotted as 1, data points with pure cultures in the placenta, but not in maternal organs, were excluded. All of the excluded data points (6/46) had a predominance of the same strain in all organs.

The strong increase of bacteria in the placenta could be due to increased replication, decreased clearance, or influx of L. monocytogenes from maternal organs to the placenta. We examined the role of trafficking of L. monocytogenes from maternal organs to the placenta in experiments with two L. monocytogenes wild-type strains, which are equal in virulence but can be distinguished by their susceptibility to erythromycin [19]. In animals infected with a 1:1 ratio of both strains, the ratio should remain 1 in organs seeded with a high number of bacteria because neither strain has a growth advantage over the other. On the other hand, organs seeded with a small number of bacteria would exhibit a wide range of ratios between animals, because the chances that these organs are initially seeded with a 1:1 ratio in all animals would be small. Interestingly, we found pure cultures of either the erythromycin-sensitive (erm^S^) or erythromycin-resistant (erm^R^) strain in 13/14 placentas 24 h after infection with a 1:1 ratio of the two wild-type strains (Figure 3). A mixed culture with a 10^2^-fold predominance of the erm^R^ strain was found in one placenta. A higher i.v. inoculum led to predominantly mixed infections (unpublished data). These results followed Poisson statistics almost perfectly. Our data suggests that 13/14 cases of placental infection were clonal in origin and led us to conclude that a single bacterium is sufficient to cause placental infection. This is consistent with reports that placental infection with L. monocytogenes in the pregnant mouse model is initiated by very low numbers or even a single organism [17,20].

At later time points during infection, we found an increase in mixed cultures in the placenta, until 21/23 placental cultures contained a mixture of both strains at 72 h p.i. (Figure 3). Thus, L. monocytogenes traffics from maternal organs to the placenta. In order to estimate the degree of influx to the placenta, we compared CFUs per placenta with changes in ratio occurring between 24 and 48 h after inoculation. At 24 h p.i. the infected placentas contained a range of 10–10^5^ CFUs (Figure 1). To change a ratio of 100 in a pool of 10^3^ bacteria to a ratio of 1 for example, influx of 10^2^ bacteria is required. On the other hand, to change a pure culture of ten bacteria to a ratio of 10, influx of only one bacterium is required. Considering all possible combinations of observed CFUs per organ (Figure 1) and changes in ratio (Figure 3), we estimated that fewer than ten bacteria traffic to the placenta between 24 and 48 h p.i. Thus, the vast majority of bacteria in the placenta was due to bacterial growth and not to influx from maternal organs. Therefore, the placenta was fairly protected from colonization throughout the entire course of the infection.

### Mathematical Model for Bacterial Growth, Killing, and Movement in an Animal

**Figure 4:**
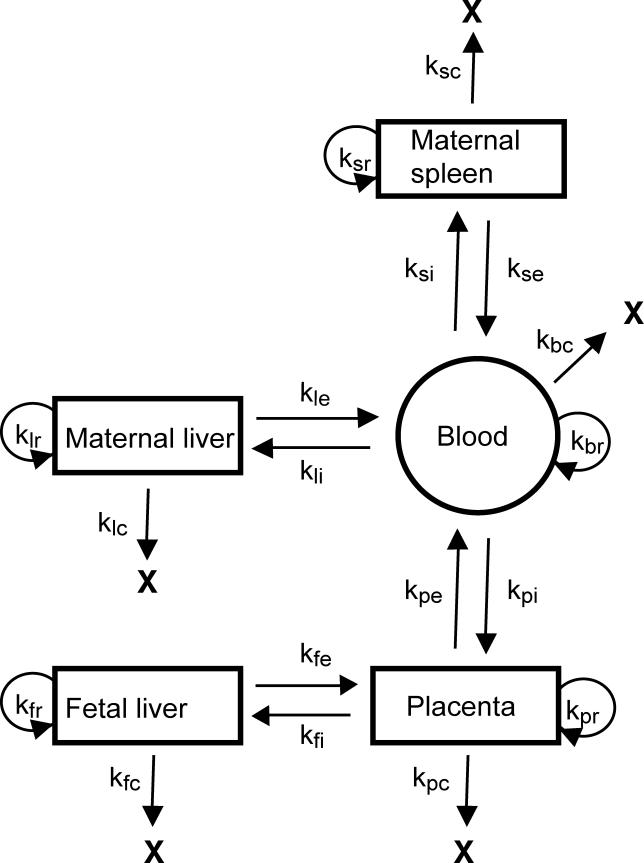
Mathematical Model for Growth, Killing, and Movement in an Infected Animal Maternal liver (l), spleen (s), and placenta (p) are separate compartments connected by the maternal bloodstream (b), treated as a fourth compartment. The fetal liver (f), the fifth compartment, is connected to the placenta. Within each of the five compartments, the number of bacteria can change by four processes: replication (r), clearance (c), efflux to another compartment (e), and influx from another compartment (i). The connections among the compartments and the relevant first-order rate constants (k) are diagrammed.

The bacterial increase in the placenta might be due to enhanced bacterial replication or decreased clearance in the placental environment. Furthermore, the difference in the course of infection between pregnant and nonpregnant animals may be due to (a) systemic suppression of the maternal immune system leading to an increase in bacterial load in maternal organs and placenta, or (b) unrestricted bacterial growth in the protected compartment of the placenta with subsequent seeding of maternal organs without differences in the systemic maternal immune response. In order to differentiate between these possibilities, we constructed a simple mathematical model for bacterial growth, killing, and movement among organs in an infected animal (Figure 4). In this model the number of bacteria in each organ was dependent on initial seeding, bacterial replication, clearance, influx, and efflux. The model allowed us to make predictions about bacterial growth, clearance, and trafficking between different organs, which subsequently were tested in the pregnant guinea pig model.

The change of bacteria over time in maternal liver, spleen, blood, placenta, and fetal liver was simulated over a 72-h course of infection. The quantitative output of the simple first-order numerical simulation showed the following striking features consistent with the experimental data. For nonpregnant animals, bacteria were slowly cleared from the spleen and remained at a nearly constant level in the liver, while in the bloodstream the numbers of bacteria remained below the detection limit (Figure 2).

For the pregnant animals, bacterial colonization of the placenta was extremely inefficient, such that on average only one bacterium colonized the placenta in the first 5 h of infection, an average rate that was 10^4^-fold less than the colonization rate into the maternal liver (Figure 1). Despite this very low rate of influx into the placenta, the low rate of bacterial clearance in the placental compartment allowed rapid net increase in the number of bacteria present. The relatively low CFUs in the fetal livers and their delay in colonization suggest that spread of bacteria from placenta to fetus is also an extremely inefficient step, on the order of 10^4^-fold less than placental colonization from the maternal bloodstream (this was true regardless of the postulated rates of bacterial replication, clearance, and efflux in the fetal liver) (Figure 1). By about 40–50 h p.i., the number of bacteria in the placenta, as well as maternal blood and organs, rose (Figure 1). In order to achieve this result, the rate of bacterial efflux from the placental compartment back into the maternal bloodstream did not need to be particularly high; rates comparable to the efflux rates for the maternal liver and spleen were fully sufficient to cause this outcome.

**Figure 5:**
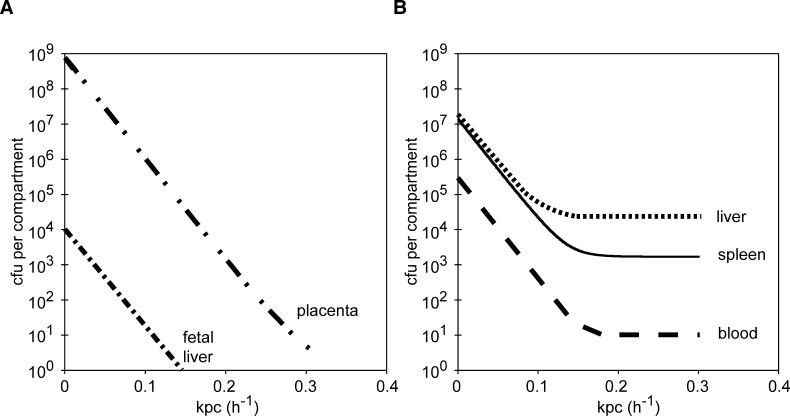
Computational Simulation of Bacterial Load in Relation to Clearance Rate in the Placenta Simulation of CFUs per (A) placenta and fetal liver and (B) maternal liver, spleen, and blood at 72 h as a function of the placental clearance rate (k_pc_).

What special feature of the placental compartment altered the overall course of infection in pregnant animals? As detailed above, the placenta was fairly refractory to bacterial colonization, so placental susceptibility to infection is not the explanation. According to the numerical simulation, the most important variable was the rate of bacterial clearance relative to the rate of bacterial replication in the placenta. Holding the rate of bacterial replication at a constant value of 0.3 h^−1^ (comparable to the replication rate in the maternal liver of 0.33 h^−1^), we determined the final number of bacteria expected 72 h after inoculation as a function of the postulated value of the placental clearance rate k_pc_ (Figure 5). In the simulation, any increase in the placental clearance rate above 0 had a strong effect on the net outcome of the infection in all organs. For postulated values of the placental clearance rate k_pc_ above 0.2 h^−1^, comparable to the clearance rates for the maternal liver and spleen of 0.33 and 0.27 h^−1^, respectively, the final bacterial load in maternal liver, spleen, and bloodstream at 72 h p.i. would be indistinguishable from that of a nonpregnant animal, and the fetus would not be infected.

It is likely, that in vivo, the rate constants of the biological processes we were trying to simulate change over time. This is probably the cause of observed differences between the mathematical simulation and the experimental data; for example, the shape of the splenic growth curve (Figure 1D). Because it is impossible to confirm changes in the rate constants experimentally, we assumed that all the processes were first order and found that the mathematical model was consistent enough with the experimental data to make the following predictions: (a) trafficking of L. monocytogenes from the placenta to maternal organs is sufficient to explain the differences in listeriosis in pregnant versus nonpregnant animals, and (b) the bacterial loads in placenta and maternal organs correlate.

### L. monocytogenes Traffics from the Placenta Back to Maternal Organs

In pregnant guinea pigs inoculated with a 1:1 ratio of two equally virulent L. monocytogenes strains, the ratios in maternal liver and spleen were 1 at 24 h p.i. (Figure 3B). Surprisingly, the ratio between the two strains became skewed at 48 h and was found to be around 1 again at 72 h (Figure 3B). In contrast, the ratio between both strains in liver and spleen remained 1 at all time points in nonpregnant animals (Figure 3C). Furthermore, when we plotted the ratios of pooled placental ratios over maternal organ ratio for each animal, we found skewed ratios at 24 h and ratios around 1 at 48 and 72 h p.i. (Figure 3E). These observations suggest that the increase in the bacterial burden in maternal liver, spleen, and bacteremia originate from the placenta, and were consistent with trafficking of L. monocytogenes from the placenta back to maternal organs. Trafficking to maternal organs must occur in large numbers because >10^5^ bacteria are necessary to change a ratio of 1:1 in a pool of 10^5^ bacteria.

### Course of Infection in Animals Treated with Gentamicin

**Figure 6:**
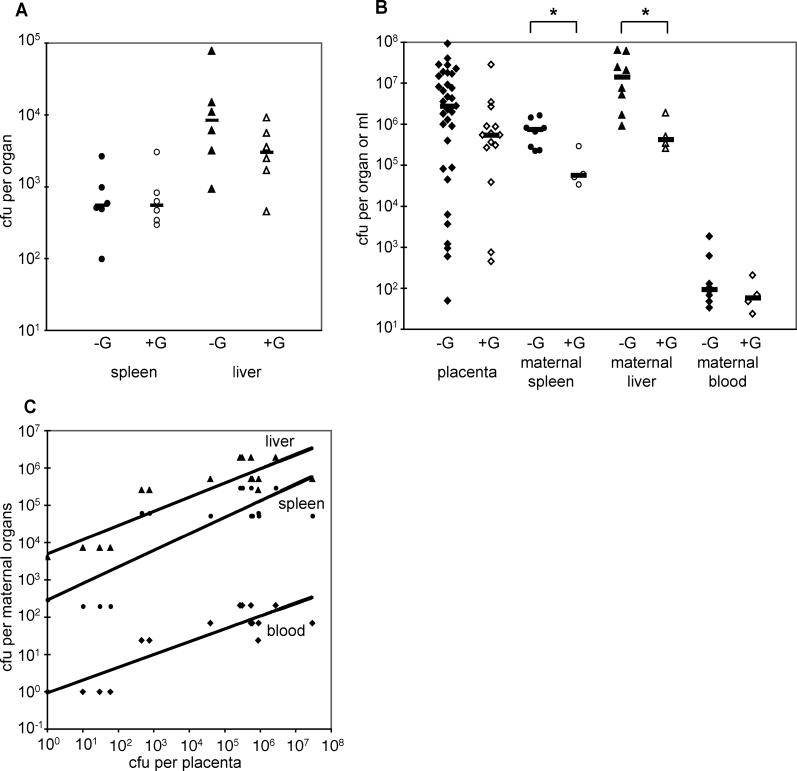
Listeriosis in Animals Treated with Gentamicin (A) CFUs per spleen and liver in nonpregnant animals were enumerated at 72 hours p.i. with 7.5 × 10^5^–10^6^ L. monocytogenes. Animals treated with gentamicin (+G) are represented by hollow symbols, untreated control animals (−G) are represented by solid symbols. Black bars represent median values. *P*-values between treated and control animals are not statistically significant (0.18 and 0.94 for liver and spleen, respectively). (B) CFUs per placenta, maternal spleen, liver, and ml of blood from pregnant animals with placental infection were enumerated at 72 hours p.i. with 7.5 × 10^5^–10^6^ L. monocytogenes. *P*-values marked with * between treated and control animals were statistically significant (0.028 and 0.016 for liver and spleen, respectively). (C) CFUs per maternal liver, spleen, and blood were plotted against CFUs per placenta. The black lines represent the best-fit power law curve for liver (y = 4818^0.38^; R^2^ = 0.85), spleen (y = 278.7^0.44^; R^2^ = 0.78), and blood (y = 0.907^0.34^; R^2^ = 0.84).

We tested the prediction that the bacterial load in the placenta is the major factor determining the seeding of maternal organs by examining the outcome of infection in animals under constant delivery of the antibiotic gentamicin. Gentamicin kills extracellular L. monocytogenes without affecting intracellular bacteria in cell cultures [21,22]. In nonpregnant mice and guinea pigs, gentamicin has no effect on the bacterial burden in the spleen [23] (Figure 6A). In the liver, gentamicin leads to a 10-fold reduction in bacterial CFUs in mice [23] and a 3-fold decrease in guinea pigs (Figure 6A), which was statistically not significant (*p*-value, 0.18). We delivered gentamicin via continuous infusion into the subcutaneous tissue utilizing osmotic pumps. Animals implanted with osmotic pumps filled with normal saline did not show any differences in bacterial burden of liver and spleen in comparison to animals without any implanted pumps (unpublished data).

In the placenta many bacteria are extracellular [12,18]. Thus, we reasoned that treatment of infected pregnant animals with gentamicin would affect the clearance rate in the placenta more than the clearance rate in maternal organs. Gentamicin treatment decreased the final load in the placenta on average by about 10-fold (Figure 6B). According to the plot in Figure 5A, this is equivalent to increasing the rate of bacterial clearance relative to replication in the placental compartment by about 0.04 h^−1^, and should also cause an approximate 10-fold decrease in the bacterial load in maternal organs. Indeed, we found 33- and 13-fold lower CFUs in maternal liver (*p*-value, 0.016) and spleen (*p*-value, 0.028), respectively, in animals treated with gentamicin in comparison to untreated animals at 72 h p.i. (Figure 6B). Furthermore, when CFUs at 72 h were plotted in all the organs as functions of each other, the numbers in maternal organs followed a power law with respect to the placental load, with an exponent of about 0.4 in all cases (Figure 6C). This was not the case when CFUs were plotted as a function of the load in any other organ (unpublished data), consistent with our hypothesis that the bacterial load in the placenta is the major factor determining the influx to maternal organs.

The observation that the bacterial load in placenta and maternal organs correlated in the presence of gentamicin suggests that trafficking of L. monocytogenes between placenta and maternal organs occurs in a compartment protected from the effects of gentamicin. Thus, it is likely that trafficking occurs inside of cells, because gentamicin kills extracellular but not intracellular L. monocytogenes. This would also be consistent with the non-significant decrease in CFUs we observed in the bloodstream of gentamicin-treated animals (Figure 6B). Interestingly, infected maternal monocytes have been shown to be involved in causing meningoencephalitis in mice [21]. The role of infected maternal cells in placento-fetal listeriosis and the question whether L. monocytogenes utilizes monocytes or macrophages, specifically for dissemination in the host, is of great interest and remains to be fully answered.

### A New Model for the Pathogenesis of Listeriosis during Pregnancy

In conclusion, tropism of L. monocytogenes to the placenta and systemic maternal cell-mediated immunosuppression have been postulated to cause the observed increase in susceptibility to listeriosis during pregnancy [6,24], despite lack of experimental evidence in vivo. Our studies in the pregnant guinea pig suggest that neither of these mechanisms play a role in placento-fetal listeriosis. Previous observations in the pregnant guinea pig model with a L. monocytogenes mutant, unable to replicate in the host, suggested that lack of effective bacterial clearance in the placenta might play a role [18]. In this study, we examined the kinetics of movement, replication, and clearance of L. monocytogenes in the guinea pig. Our data is consistent with a new model for the pathogenesis of listeriosis during pregnancy where the placenta is relatively protected from colonization. However, once infected, the placenta acts as a nidus of infection for the mother. In the natural course of infection, seeding of maternal organs will eventually be interrupted by expulsion of the infected placenta. Therefore, spontaneous abortion and preterm labor can be regarded as survival mechanisms for the mother**.** Our conclusions are entirely supported by the experimental data presented in this paper. However, the mathematical model helped to clarify the kinetics of movement and emphasized the importance of decreased clearance in bacterial burden of the placenta. The mathematical model can be refined further and used in the future to ask other questions about this system: for example, dissemination of L. monocytogenes from the intestine, the role of specific immune-effector cells (e.g., macrophages in trafficking), or the effect of cytokines on bacterial clearance [25].

Another interesting aspect of our findings is the implication for transmission in the natural setting of listeriosis. Many mammals, for example, cattle, develop spontaneous abortions secondary to infection with L. monocytogenes [26]. In this setting, the aborted placenta, which contains very high numbers of *L. monocytogenes,* will contaminate the environment and the food supply of other animals enabling L. monocytogenes to spread efficiently to other hosts, similar to the transmission of other pathogens [27].

## Materials and Methods

### Bacteria.

*L. monocytogenes* strains used in this study were 10403S (erm^S^) [28] and DP-L3903 (erm^R^) [19]. Bacteria for in vivo infections were prepared as described previously [12].

### Animals.

All animals were housed and handled in accordance with federal and institutional guidelines. The animal use committee at the University of California, Berkeley approved the animal use protocol describing our studies. Nonpregnant or pregnant female Hartley guinea pigs (gestational age 25 d) were purchased from Elm Hill Breeding Labs. Animals were injected i.v. with a 1:1 ratio of 10403S and DP-L3903 at a total dose of 7.5 × 10^5^ −10^6^ bacteria on day 39 of gestation as described previously [18]. At specified time points, animals were placed under general anesthesia and euthanized after blood was drawn by cardiac puncture. Blood was diluted 1:1 with 0.2% NP-40 (Biosciences, Incorporated) and sonicated for 10 s. CFUs in 1 ml of blood (detection limit ~10 CFUs per animal) and each entire placenta (on average 3–4 placentas per animal) were determined. CFUs per maternal liver, spleen, and fetal liver were determined as described previously [12]. A minimum of 100 CFUs per organ (or all colonies if <100) were patched onto agar plates containing erythromycin (Sigma, St. Louis, Missouri, United States) at a concentration of 2 μg/ml.

### Implantation of osmotic pumps.

Alzet osmotic pumps (model 2ML1, release rate 10 μl/h, Durcet Corporation, Cupertino, California, United States) were filled with injection grade gentamicin sulfate at 100 mg/ml (Phoenix Scientific, St. Joseph, Missouri, United States) and incubated overnight in 0.9% sterile sodium chloride solution at 37 °C. Guinea pigs were placed under general anesthesia 24 h after bacterial inoculation, fur was shaved on the back between the shoulder blades, and the skin was cleaned with chlorhexidine scrub and 70% ethanol. Local anesthetic (2% lidocaine, Abbott Laboratories, Abbott Park, Illinois, United States) was injected subcutaneously and a horizontal incision was made. Using a hemostat, a small pocket was formed by spreading the subcutaneous connective tissues apart. The pump was inserted into the pocket and the skin was closed with wound clips.

### Determination of serum gentamicin levels, inhibitory, and bactericidal titers.

Blood was drawn by venipuncture at 2, 4, 20, 24, 48, and 72 h after implantation of osmotic pumps in three animals and prior to euthanization in all other animals. Serum was collected from blood by centrifugation for 20 min at 6,000 rpm at 4 °C. Gentamicin levels were determined at the Veterinary Medical Teaching Hospital at the University of California, Davis and were all approximately 5 μg/ml (range 3.2–6.8 μg/ml). Minimum inhibitory and bactericidal titers were determined as previously described [21] and were <0.6 μg/ml and 1.25–2.5 μg/ml, respectively, consistent with previous reports [29].

### Construction of mathematical model for bacterial growth, killing, and movement.

The probable connections between maternal organs and blood, placenta, and fetal liver are diagrammed in Figure 4. Within each compartment, the number of bacteria as a function of time was determined by the initial number of bacteria and the quantitative contribution of replication, clearance, influx, and efflux. We assumed that all the first-order rate constants remain fixed throughout the course of the infection. For example, the change in the number of bacteria in the liver (L) as a function of time was given by: dL/dt = k_lr_*L − k_lc_*L − k_le_*L + k_li_*B, where k_lr_ is the replication rate for bacteria in the liver, k_lc_ is the clearance rate for bacteria in the liver, k_le_ is the efflux rate for bacteria to exit the liver and enter the bloodstream, k_li_ is the influx rate for bacteria to leave the blood and enter the liver, and (B) is the number of bacteria in the bloodstream compartment. Accordingly, the set of differential equations for the remaining compartments was as follows:


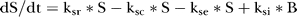



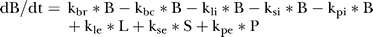



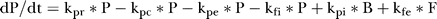



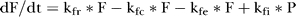


The real values for several of the rate constants for the guinea pig model of listeriosis were deduced or estimated from the quantitative experiments described above and other experiments in the guinea pig model previously published [18]. Clearance rates for placenta (k_pc_), maternal liver (k_lc_), and spleen (k_sc_) were based on inoculation of pregnant guinea pigs with 10^9^ CFUs of the LLO-minus mutant DP-L2161. Replication rates in the maternal organs were estimated by examining the kinetics of bacterial infection in liver and spleen for nonpregnant animals. In these animals, no bacteria were detectable in the bloodstream at any time after the initial inoculation, so the bacterial load in the organs must have been primarily determined by the replication and clearance rates, with influx and efflux making very minor contributions. Using the clearance rates as determined above, replication rates for the maternal organs (k_lr_, k_sr_) were calculated. Influx rates into maternal liver (k_li_) and spleen (k_si_), placenta (k_pi_), and fetal liver (k_fi_) were estimated by examining the initial colonization efficiency of these organs after inoculation of animals with a high dose of bacteria. In the nonpregnant animals the placental influx rate (k_pi_) was set as 0 with all other rate constants left unchanged. The remainder of the rate constants for placental replication k_pr_, efflux rates from maternal organs and placenta (k_le_, k_se_, k_pe_), clearance rate in the maternal blood k_bc_, and fetal rate constants (k_fr_, k_fc_, k_fe_) were determined by fitting the simulation to the experimental data with Berkeley Madonna [30].

Values of all rate constants used in the numerical simulation are tabulated here. Equations were integrated using Berkeley Madonna [30].


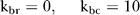



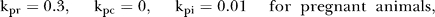



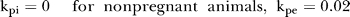



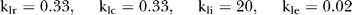



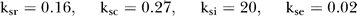



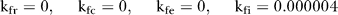


### Statistical analysis.

*P*-values were determined with the Mann-Whitney test: http://www.statpages.org.

## Supporting Information

Figure S1 Bacterial Load in Pregnant Animals without Placental Infection CFUs per maternal spleen, liver, and ml of blood from pregnant animals without placental infection at 72 h post i.v. inoculation with 7.5 × 10^5^–10^6^ L. monocytogenes were enumerated.
(187 KB PDF)

## Abbreviations

CFU: colony forming unit
erm^S^: erythromycin sensitive
erm^R^: erythromycin resistant
i.v.: intravenous
p.i.: post-inoculation
