# Correction: Listeria monocytogenes Traffics from Maternal Organs to the Placenta and Back

**DOI:** 10.1371/journal.ppat.0020080

**Published:** 2006-07-28

**Authors:** Anna I Bakardjiev, Julie A Theriot, Daniel A Portnoy

In *PLoS Pathogens*, volume 2, issue 6: DOI: DOI: 10.1371/journal.ppat.0020066

The PDF version of the above article states that a previous version was published as an Early Online Release on June 23, 2006. This information appeared in error. No previous version of the article has been published.

