# Supplementary figures and images for: Listeria monocytogenes Traffics from Maternal Organs to the Placenta and Back
Source: PLoS Pathog. 2006 Jun 30;2(6):e66. doi: 10.1371/journal.ppat.0020066 (PMC1483233; doi:10.1371/journal.ppat.0020066)

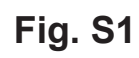

**Fig. S1**

Supplement: Figure S1 — CFUs per maternal spleen, liver, and ml of blood from pregnant animals without placental infection at 72 h post i.v. inoculation with 7.5 × 10^5^–106 L. monocytogenes were enumerated. (187 KB PDF) [file ppat.0020066.sg001.pdf]
